# A169 THE DIRECT COSTS OF INFLAMMATORY BOWEL DISEASE IN CANADA: A POPULATION-BASED ANALYSIS OF HISTORICAL AND CURRENT COSTS

**DOI:** 10.1093/jcag/gwac036.169

**Published:** 2023-03-07

**Authors:** S Coward, E I Benchimol, C Bernstein, J A Avina-Zubieta, A Bitton, L Hracs, J Jones, E Kuenzig, L Lu, S K Murthy, Z Nugent, A R Otley, R Panaccione, J -N Pena-Sanchez, H Singh, L E Targownik, J W Windsor, G Kaplan

**Affiliations:** 1 University of Calgary, Calgary; 2 The Hospital for Sick Children, Toronto; 3 University of Manitoba, Winnipeg; 4 University of British Columbia, Vancouver; 5 McGill University, Montreal; 6 Dalhousie University, Halifax; 7 Arthritis Research Canada, Vancouver; 8 The Ottawa Hospital, Ottawa; 9 University of Saskatchewan, Saskatoon; 10 University of Toronto, Toronto, Canada

## Abstract

**Background:**

Inflammatory bowel disease (IBD) is a costly disease to manage due to hospitalizations, regular ambulatory monitoring, and expensive pharmaceutical therapies. While hospitalization rates have fallen, the increased use of biologics have escalated the cost of care to the healthcare system.

**Purpose:**

To assess historical direct healthcare costs of the IBD population in Canada.

**Method:**

Population-based administrative costing data were obtained from: Alberta, British Columbia, and Manitoba. Costs were calculated based on administrative data (2009 to 2016) which captured: hospitalizations, physician costs, ambulatory care such as: emergency visits, day surgery, and colonoscopy (AB only), and medication costs of IBD-specific medications, such as: mesalamine, biologics, steroids, and immunomodulators. Costs were converted to 2020 dollars using the consumer price index. Average annual cost per person (ACPP) was calculated for each province. Using province specific IBD prevalence estimates these ACPP were meta-analyzed to obtain the annual weighted costs, with 95% confidence intervals (CI), and these costs underwent meta-regression to ascertain the average annual change in cost per year. An Autoregressive Integrated Moving Average model was created to estimate the ACPP in 2023 with 95% prediction intervals (PI). Canada-wide total direct care costs of IBD patients, in billions (B), were calculated using the ACPP, Canada-specific IBD prevalence estimates (historical and forecasted), and total Canadian population calculations from Statistics Canada (historical and forecasted).

**Result(s):**

In 2009 the ACPP was $7000 (95%CI: 5389, 8610), representing $1.18B (95%CI: 0.91B, 1.45B) in direct healthcare costs in Canada for all IBD patients. The ACPP in 2016 was increased to $10,336 (95%CI: 6803, 13869), which equates to $2.37B (95%CI: 1.56B, 3.18B) per year in direct healthcare costs. From 2009 to 2016, the ACPP increased an average of $450 (95%CI: 132, 767) per year. If these historical trends continue to 2023 the ACPP is forecasted to be $13,333 (95%PI: 12827, 13839) per person per year. The largest contributor to these costs is medications—accounting for an estimated 50% of the total costs of IBD patients.

**Image:**

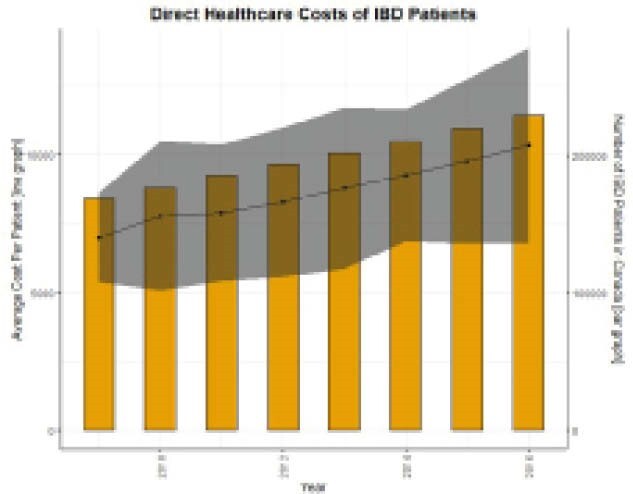

**Conclusion(s):**

The direct healthcare cost of IBD has risen steadily from 2009 to 2016 when the healthcare system spent over $10,000 per person with IBD and $2.37B nationwide. The primary driver of costs is medical management. Forecast models estimate that the annual cost may be over $13,000 per person in 2023. However, these estimates do not account for advent and increased uptake of novel biologics and small molecules, nor the downward cost pressure of biosimilars. These costs are those paid directly by the healthcare system and do not account for those born by the individual—it is estimated that the true cost of IBD (direct and indirect) is much higher.

**Please acknowledge all funding agencies by checking the applicable boxes below:**

CIHR

**Disclosure of Interest:**

S. Coward: None Declared, E. Benchimol Consultant of: Hoffman La-Roche Limited and Peabody & Arnold LLP for matters unrelated to medications used to treat inflammatory bowel disease and McKesson Canada and the Dairy Farmers of Ontario for matters unrelated to medications used to treat inflammatory bowel disease., C. Bernstein Grant / Research support from: Unrestricted educational grants from Abbvie Canada, Janssen Canada, Pfizer Canada, Bristol Myers Squibb Canada, and Takeda Canada. Has received research grants from Abbvie Canada, Amgen Canada, Pfizer Canada, and Sandoz Canada and contract grants from Janssen, Abbvie and Pfizer, Consultant of: Abbvie Canada, Amgen Canada, Bristol Myers Squibb Canada, JAMP Pharmaceuticals, Janssen Canada, Pfizer Canada, Sandoz Canada, and Takeda., Speakers bureau of: Abbvie Canada, Janssen Canada, Pfizer Canada and Takeda Canada, J. A. Avina-Zubieta: None Declared, A. Bitton: None Declared, L. Hracs: None Declared, J. Jones Consultant of: Janssen, Abbvie, Pfizer, Takeda, Speakers bureau of: Janssen, Abbvie, Pfizer, Takeda, E. Kuenzig: None Declared, L. Lu: None Declared, S. Murthy: None Declared, Z. Nugent: None Declared, A. Otley Grant / Research support from: Unrestricted educational grants from AbbVie Canada and Janssen Canada, Consultant of: Advisory boards of AbbVie Canada, Janssen Canada and Nestle, R. Panaccione Consultant of: Abbott, AbbVie, Alimentiv (formerly Robarts), Amgen, Arena Pharmaceuticals, AstraZeneca, Biogen, Boehringer Ingelheim, Bristol-Myers Squibb, Celgene, Celltrion, Cosmos Pharmaceuticals, Eisai, Elan, Eli Lilly, Ferring, Galapagos, Fresenius Kabi, Genentech, Gilead Sciences, Glaxo-Smith Kline, JAMP Bio, Janssen, Merck, Mylan, Novartis, Oppilan Pharma, Organon, Pandion Pharma, Pendopharm, Pfizer, Progenity, Protagonist Therapeutics, Roche, Sandoz, Satisfai Health, Shire, Sublimity Therapeutics, Takeda Pharmaceuticals, Theravance Biopharma, Trellus, Viatris, UCB. Advisory Boards for: AbbVie, Alimentiv (formerly Robarts), Amgen, Arena Pharmaceuticals, AstraZeneca, Biogen, Boehringer Ingelheim, Bristol-Myers Squibb, Celgene, Eli Lilly, Ferring, Fresenius Kabi, Genentech, Gilead Sciences, Glaxo-Smith Kline, JAMP Bio, Janssen, Merck, Mylan, Novartis, Oppilan Pharma, Organon, Pandion Pharma, Pfizer, Progenity, Protagonist Therapeutics, Roche, Sandoz Shire, Sublimity Therapeutics, Takeda Pharmaceuticals, Speakers bureau of: AbbVie, Amgen, Arena Pharmaceuticals, Bristol-Myers Squibb, Celgene, Eli Lilly, Ferring, Fresenius Kabi, Gilead Sciences, Janssen, Merck, Organon, Pfizer, Roche, Sandoz, Shire, Takeda Pharmaceuticals, J.-N. Pena-Sanchez: None Declared, H. Singh Consultant of: Pendopharm, Amgen Canada, Bristol Myers Squibb Canada, Roche Canada, Sandoz Canada, Takeda Canada, and Guardant Health, Inc.,, L. Targownik Grant / Research support from: Investigator initiated funding from Janssen Canada, Consultant of: [Advisory board] AbbVie Canada, Takeda Canada, Merck Canada, Pfizer Canada, Janssen Canada, Roche Canada, and Sandoz Canada, J. Windsor: None Declared, G. Kaplan Grant / Research support from: Ferring, Janssen, AbbVie, GlaxoSmith Kline, Merck, and Shire, Consultant of: Gilead, Speakers bureau of: AbbVie, Janssen, Pfizer, Amgen, and Takeda

